# The Functional Characterization of a Site-Specific Apigenin 4′-*O*-methyltransferase Synthesized by the Liverwort Species *Plagiochasma appendiculatum*

**DOI:** 10.3390/molecules22050759

**Published:** 2017-05-07

**Authors:** Hui Liu, Rui-Xue Xu, Shuai Gao, Ai-Xia Cheng

**Affiliations:** Key Laboratory of Chemical Biology of Natural Products, Ministry of Education, School of Pharmaceutical Sciences, Shandong University, Jinan 250012, China; ttklliuhui@163.com (H.L.); xurx123@163.com (R.-X.X.); gaoshuai.3331166@163.com (S.G.)

**Keywords:** *Plagiochasma appendiculatum*, Class II *O*-methyltransferase, apigenin, acacetin, feeding

## Abstract

Apigenin, a widely distributed flavone, exhibits excellent antioxidant, anti-inflammatory, and antitumor properties. In addition, the methylation of apigenin is generally considered to result in better absorption and greatly increased bioavailability. Here, four putative Class II methyltransferase genes were identified from the transcriptome sequences generated from the liverwort species *Plagiochasma appendiculatum*. Each was heterologously expressed as a His-fusion protein in *Escherichia coli* and their methylation activity against apigenin was tested. One of the four Class II OMT enzymes named 4′-*O*-methyltransferase (Pa4′OMT) was shown to react effectively with apigenin, catalyzing its conversion to acacetin. Besides the favorite substrate apigenin, the recombinant PaF4′OMT was shown to catalyze luteolin, naringenin, kaempferol, quercetin, genistein, scutellarein, and genkwanin to the corresponding 4′-methylation products. In vivo feeding experiments indicated that PaF4′OMT could convert apigenin to acacetin efficiently in *E. coli* and approximately 88.8 µM (25.2 mg/L) of product was synthesized when 100 µM of apigenin was supplemented. This is the first time that a Class II plant *O*-methyltransferase has been characterized in liverworts.

## 1. Introduction

Flavonoids are a family composed of various polyphenolic compounds synthesized by plant species. Among this family, apigenin (4,5,7-trihydroxyflavone) is a widely distributed flavone and exhibits antioxidant, anti-inflammatory, and antitumor properties [1,2,3,4]. It was found that methylation of the free hydroxy groups in the flavonoids dramatically increases their stability and enhances the membrane transport, resulting in better absorption and greatly increased bioavailability [5,6]. Acacetin, 4′-methylated form of apigenin, has been proven to have broad-spectrum biological activities and has gradually been used in the treatment of multifarious diseases. A previous study revealed that acacetin is a promising atrium-selective agent for the treatment of anti-atrial fibrillation [7] and has been well documented to show novel pharmacological properties such as antinociceptive, anti-inflammatory, and anti-cancer activities [8,9,10]. Enzyme-catalyzed synthesis of acacetin can be achieved by the para-*O*-methylation of apigenin using *S*-adenosyl-l-methionine as a methyl donor catalyzed by *O*-methyltransferases (OMTs). *O*-methyltransferases are known to be involved in the reaction transforming methyl to hydroxy groups of phenylpropanoids and flavonoids. Based on their size, amino acid sequence, and cation dependency, they have been classified into two major groups, referred to as Classes I and II. Class IOMTs (caffeoyl-coenzyme A *O*-methyltransferases; CCoAOMT) with a molecular weight of 26–29 KDa methylate the lignin precursors caffeoyl-coenzyme A (CCoA) and 5-hydroxyferuloyl CoA in the presence of an Mg^2+^ cation premise. Substrates preferred by CCoAOMT possess two vicinal hydroxyl groups and their methylation affects the meta*-*hydroxyl group. COMTs (caffeic acid *O*-methyltransferases) are cation-independent enzymes capable of methylating not only flavonoids, but also both 3-hydroxyl-and 5-hydroxyl-containing phenylpropanoid-derived lignin precursors [11]. Class II OMTs’ molecular weights range from 38 to 43 KDa, and their activity does not require the presence of a metal cation. Besides the typical COMT activity of catalyzing the methylation of the lignin precursors caffeic acid and 5-hydroxy coniferic acid, some Class II OMTs also play a role in the methylation of flavonoids. For instance, the *Arabidopsis thaliana*enzyme AtCOMT1 is able to methylate the flavonol quercetin, which bears an orthodihydroxy group, while the *Populus deltoides* enzyme POMT-7 transfers a methyl group to flavonoids (apigenin, kaempferol, luteolin, and quercetin) to the C-7 OH group [12].

In recent years, metabolic engineering containing OMT genes has been one of the hottest fields in terms of use for the production of valuable medical compounds, especially for polyhydroxyl molecules. *O*-methylation of a specific OH group through chemical synthesis confronts many drawbacks: harsh catalytic conditions, a long reaction time, protection of unwanted groups, and expensive methylation reagents. For the production of acacetin, direct C-4′ methylation of apigenin is impossible by chemical means, due to the higher acidity of C-7 hydroxy group. It has been synthesized from largely available naringin via dehydrogenation (to rhoifolin) and hydrolysis of glycosidic side chain [13,14]. However, microbial biotransformation is an alternative strategy with great potential to produce novel bioactive compounds in a one-step reaction and a much milder reaction condition. Microbes have a highly tractable genetic system and favorable fermentation conditions [15]. Several studies have shown that high productivities of *O*-methylation for flavonoids are viable. SaOMT-2, isolated from *Streptomyces avermitilis* MA-4680, converted naringenin to sakuranetin in *E. coli*, which has an antifungal activity against *Magnaporthe grisea* [16].

**Figure 1:**
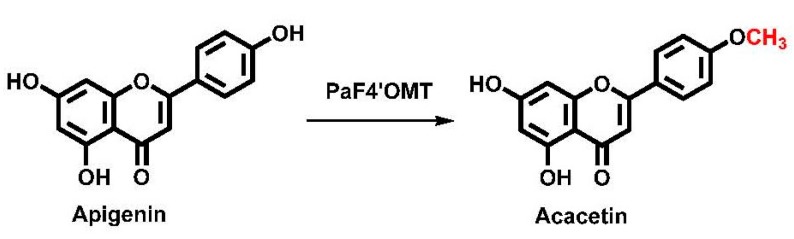
The methylation of apigenin catalyzed by 4′-*O*-methyltransferase (Pa4′OMT).

Liverworts produce methylated flavonoids, lignans, lignin-like compounds, and bis-bibenzyls. Here, a description is given of the isolation of four *Plagiochasma appendiculatum* Class II OMTs, one of which has been functionally characterized as a flavone 4′-*O*-methyltransferase (Pa4′OMT) with apigenin as the favorite substrate (Figure 1). The site-specific biotransformation of apigenin to acacetin in engineered *E. coli* expressing PaF4′OMT was explored and approximately 25.2 mg/L of acacetin was synthesized. This is the first time that a Class II plant *O*-methyltransferase, which catalyzed the *O*-methylation of apigenin to form acacetin in *E. coli*, has been characterized in liverworts.

## 2. Results

### 2.1. The PaCOMT Sequences

**Figure 2:**
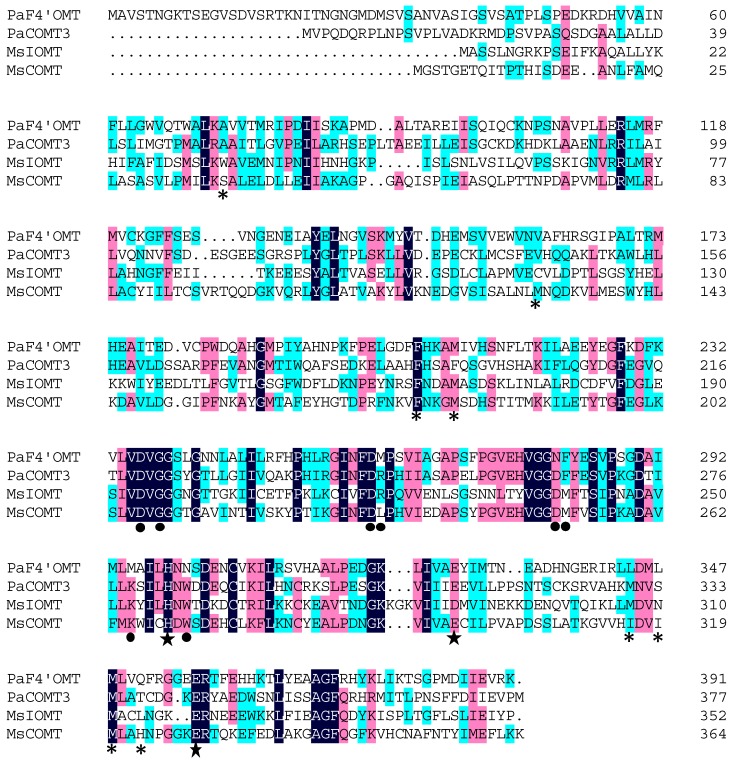
Sequence alignment of PaF4′OMT, PaCOMT3, *Medicago sativa* IOMT (MsIOMT), and *Medicago sativa* COMT (MsCOMT). Residues involved in SAM binding (●), substrate binding (*), and catalysis (★) are shown. Markers are located at the bottom of the sequences.

**Figure 3:**
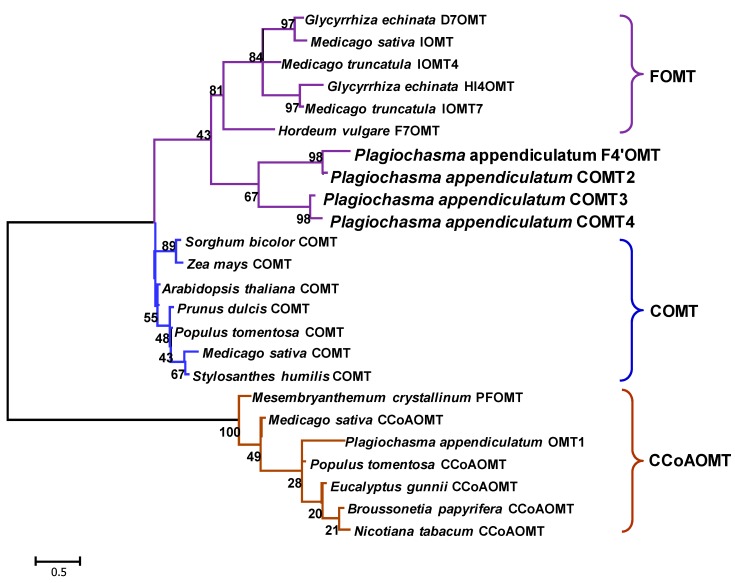
Phylogeny of the PaCOMTs and related OMTs. The scale indicates evolutionary distance. The numbers shown are bootstrap values, based on 1000 replicates. The bar represents evolutionary distance. COMT: caffeic acid *O*-methyltransferase; IOMT: isoflavonoid *O*-methyltransferase; DOMT: daidzein *O*-methyltransferase; HI4′OMT: 2,7,4′-hydroxyflavonone *O*-methyltransferase; FOMT: flavonoid *O*-methyltransferase.

Four distinct putative caffeic acid OMT unigenes were identified in the *P. appendiculatum* transcriptome, and designated as *PaCOMT1-PaCOMT4*. The 3′ end of PaCOMT2 was obtained by RACE-PCR. Amplification from *P. appendiculatum* of the four full length sequences revealed ORFs of the following lengths: 1176 bp, 1194 bp, 1137 bp, and 1137 bp, corresponding to predicted translation product sizes of 43.4 KDa, 44.1 KDa, 41.5 KDa, and 41.5 KDa, respectively. The PaCOMT1 sequence included two start codons separated by 27 amino acids. The shorter product (transcribed from the more 3′ placed ATG) was designated *PaCOMT1-Tr*, translating to a 40.7 KDa product. The peptide sequence identity between PaCOMT1 and PaCOMT2 was 68.3%, while that between PaCOMT3 and PaCOMT4 was 87.6%. A multiple sequence alignment involving PaCOMT1 and PaCOMT3 with some related proteins revealed a rather low level of identity: respectively, 31.8% and 34.3% with *Medicago sativa* COMT (MsCOMT), and 23.5% and 26.2% with *Medicago sativa* IOMT (MsIOMT) (Figure 2). The three catalytic residues (His-269, Glu-297, and Glu-329) present in MsIOMT [17] were conserved in the PaCOMTs. With respect to the SAM-binding residues, most of them were retained in PaCOMT3, but only a few in PaCOMT1. There was little evidence for the conservation of substrate-binding residues in the two PaCOMTs. A phylogenetic analysis of the PaCOMTs suggested three clades, reflecting substrate specificity (Figure 3). The Class I OMT sequences clustered together, whereas the Class IIs were distributed in a separate group. The four *PaCOMT* genes clustered with the flavone OMT genes rather than with the caffeic acid OMT genes and they were located in the root of this clade. The implication was that, unlike most COMT enzymes, the PaCOMTs likely had a substrate preference for flavonoids.

### 2.2. Purification and In Vitro Enzyme Assays of Recombinant PaCOMT1-4

**Figure 4:**
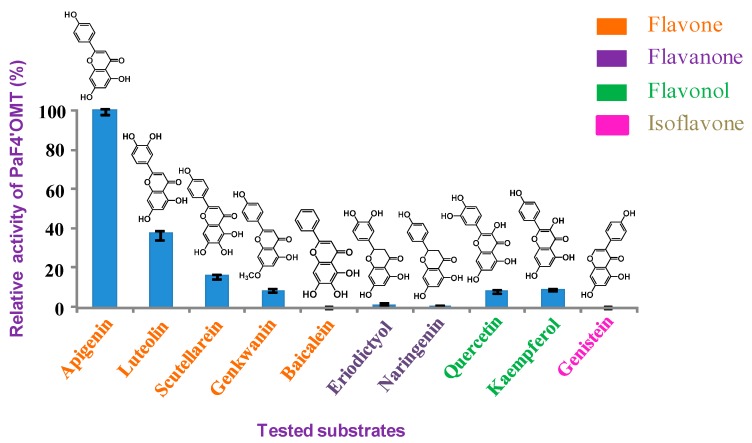
The substrate preference of PaF4′OMT. The activity towards apigenin was set at 100, and other substrates were normalized accordingly.

**Figure 5:**
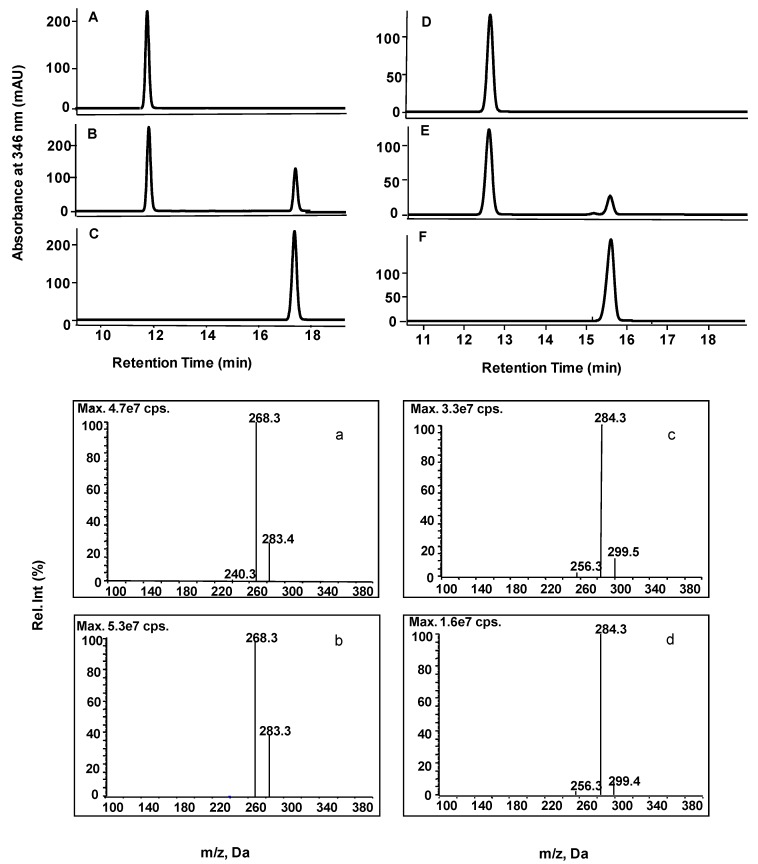
In vitro enzyme activity of recombinant proteins produced in *E. coli*, using apigenin or luteolin as the substrate. (**A**, **B**) Apigenin supplied as the substrate; HPLC analysis of reaction products of cells harboring (A) an empty vector and (B) PaF4′OMT. (**D**, **E**) Luteolin supplied as the substrate; HPLC analysis of reaction products of cells harboring (D) an empty vector and (E) PaF4′OMT. (**C**) Acacetin standard. (**F**) Diosmetin standard. The lower panel shows the mass spectrometry spectrum of (**a**, **c**) the product of PaF4′OMT supplied with (**a**) apigenin and (**c**) luteolin as substrate. (**b**) Acacetin standard. (**d**) Diosmetin standard.

When each of the PaCOMTs was expressed as a polyhistidine-tagged fusion in *E. coli,* SDS-PAGE analysis confirmed that a high level of recombinant protein expression had been achieved. The molecular mass of each of the recombinant proteins was around 60 KDa (Figure S1). As putative COMTs, caffeic acid was tested by enzyme assays, while PaCOMTs failed to convert it to ferulic acid. Among the other possible substrates, PaCOMT1 displayed activity towards the flavonoids apigenin, luteolin, scutellarein, genkwanin, naringenin, quercetin, kaempferol, and eriodictyol. The identities of all the catalytic products from the in vitro assays were confirmed by comparing the retention time and mass spectrum with their respective authentic standards. According to a standard calibration curve, the quantity of product could be calculated. Their reaction activities were summarized (Table 1, Figure 4). Its optimal substrate was apigenin (100% relative activity, 86.8 nmol/mg/min), followed by luteolin (37.8%, 32.9 nmol/mg/min) (Figure 4). The products from apigenin and luteolin shared an identical retention time as, respectively, acacetin and diosmetin. Mass spectrometry identified that the catalytic product of apigenin had a parent ion peak [M − H]^−^ at an *m/z* of 283, and among its fragmentation products was an [M – H − CH_3_]^−^ ion with an *m*/*z* of 268, consistent with acacetin. The luteolin product shared the same parent ion [M− H]^−^at an *m*/*z* of 299 and a fragment ion at an *m*/*z* of 284 as diosmetin (Figure 5). The PaCOMT1-Tr protein displayed a much lower level of substrate selectivity and activity (Table 1), suggesting that the longer PaCOMT1 sequence dominates in vivo. None of PaCOMT 2-4 displayed any sign of activity against any of the putative substrates. The conclusion was that, since PaCOMT1 was able to catalyze flavonoids to their 4′-*O*-methylether, it was admissible to rename it as PaF4′OMT. Its optimal temperature was 37°C. PaF4′OMT showed a high catalytic activity over the pH range 7.5–9.0, although the substrates became instable at pH >8 (Figure S2). The absence of Mg^2+^ had no effect on its activity, in accordance with the behavior of Class II OMTs. Under optimal conditions (37°C, pH 7.5), the enzyme’s *K_m_* was 31.0 µM and its *K_cat_* was 0.12 s^−1^ when provided with apigenin, and, respectively, 52.1 µM and 0.08 s^−1^ when provided with luteolin (Table 2), indicating that the enzyme has a stronger binding affinity and thus a higher catalytic efficiency towards apigenin than that towards luteolin in vitro.

### 2.3. Synthesis of 4′-Methylated Form of Apigenin in E. coli

**Figure 6:**
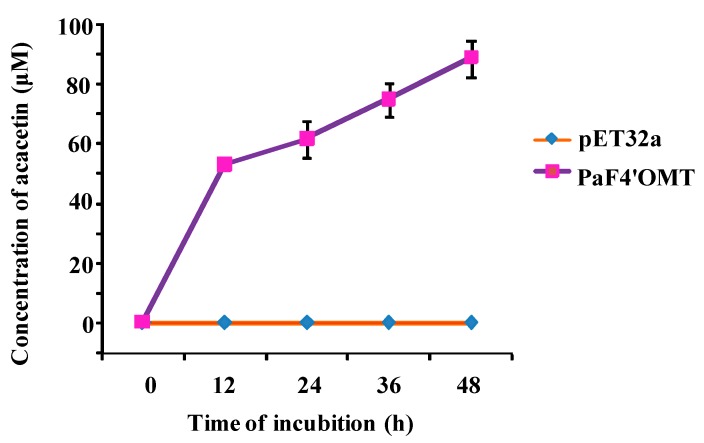
The catalysis of apigenin by PaF4′OMT in *E. coli* cells provided with 100 μM apigenin after a 48 h incubation. The control is represented by cells harboring an empty pET32a vector.

The *E. coli* strain harboring the pET32a-PaF4′OMT construct was fed with apigenin in order to produce acacetin. HPLC analysis of the reaction product confirmed that a single product was produced, as in the in vitro reactions. The conversion after 12 h was 15.0 mg/L (52.9 μM), after 24 h 17.5 mg/L (61.6 μM), after 36 h 21.3 mg/L (74.9 μM), and after 48 h 25.2 mg/L (88.8 μM) (Figure 6). An 88.8% conversion of apigenin to acacetin was achieved after the 48 h incubation, a level which is promising for the bio-based synthesis of 4′-methylated form of apigenin.

## 3. Discussion

In the present investigation, four putative COMT-encoding genes have been isolated from the liverwort species *P. appendiculatum* and expressed heterologously in *E. coli.* Three of these were unable to catalyze a range of phenylpropanoid and flavonoid substrates, and PaF4′OMT was tested to react most effectively with apigenin. Although the PaF4′OMT sequence was more like that of a COMT than a flavonoid OMT, the present experimental substrate specificity tests and kinetic analyses (Table 1 and Table 2) indicated that it was a flavonoid, B-ring-specific OMT. A similar outcome resulted from the characterization of wheat OMT2 (TaOMT2), which, inspite of its homology to the COMTs, displays a pronounced preference for a flavone substrate [18]. The TaOMT2 catalyzed three sequential *O*-methylations of a flavonoid substrate, while PaF4′OMT showed strict site-specificity to the flavonoid 4′-OH group. PaF4′OMT exhibited a high level of site-specificity and a narrow substrate recognition. Its differential response to the flavanoids naringenin and apigenin showed that its catalytic efficiency was promoted by the presence of a double bond between Positions 2 and 3. Meanwhile, the presence of a 3′-OH group impaired catalysis, since the rate of luteolin conversion was only 38% that of apigenin. A methyl group at Position 7 was not beneficial, as demonstrated by the contrasting responses to genkwanin and apigenin. Scutellarein (which carries a 4′-OH group in its B ring) was converted to hispidulin by PaF4′OMT, while baicalein was not accepted as substrate; these results further confirmed that the presence of a 4′-OH group is essential for PaF4′OMT activity. The isoflavone genistein showed the same 4′-OH group as apigenin except for the attachment position of the B ring. This slight difference was sufficient to greatly compromise enzyme activity. When provided with methyl apigenin already methylated on its A ring (Position 7), the ability of PaF4′OMT to transfer a methyl group to the 4′-OH group was inhibited.

Given the great potential application of acacetin in the field of medicine, initial researchers focused its production on plants [19,20,21]; however, these extractions were restricted by the relative low content of the metabolite in the plant. The leading outcome of the present research is the identification of a candidate gene encoding an enzyme able to synthesize acacetin from apigenin. The chemical synthesis of methylated flavonoids requires that non-target hydroxyl groups be protected, and this is assured by site-specific enzyme-based protocol. While enzymatic alkylation remains an important target of biocatalysis, it is impeded by a lack of effective SAM recycling methods. In contrast, *E. coli* has a highly tractable genetic system, gradually being one of the most widely used microorganisms for the production of natural products and their derivatives [22]. In the present investigation, a biotransformation method to produce acacetin from apigenin was introduced. The transformation system was based on engineered *E. coli* harboring PaF4′OMT as a host and exogenous addition of substrate. An 88.8% conversion of apigenin to acacetin in *E. coli* after 48 h was implied, which is promising for a method of producing acacetin without exogenous SAM. The *E. coli* system is designed to be a simple factory allowing production of acacetin in much less time and with much fewer economic materials, so this plant methyltransferase appears to act as an efficient tool to represent viable biocatalysis.

## 4. Materials and Methods

### 4.1. Plant Materials and Reagents

*P. appendiculatum* plants were maintained in a greenhouse under a 12 h photoperiod and a temperature of ~25°C. Flavonoids and their methylated products, caffeic acid, SAM, and other reagents were purchased from Chengdu Must Bio-technology (Chengdu, China), Alfa Aesar (Heysham, UK), and Sigma-Aldrich (St. Louis, MO, USA), respectively. The synthesis of caffeoyl aldehyde, caffeoyl alcohol, 5-hydroxy coniferyl aldehyde, and 5-hydroxy coniferyl alcohol was performed following procedures published elsewhere [23,24]. CCoA was synthesized from caffeic acid using liverwort 4-coumarate CoA ligase [25].

### 4.2. Sequence Analysis and cDNA Cloning

Total RNA extracted from rinsed, snap-frozen two-month-old *P. appendiculatum* thalli, and it was used as template for cDNA synthesis, based on the use of a PrimeScript™ RT Master Mix (Takara, Otsu, Japan), following the manufacturer’s protocol [26]. Four OMT sequences (*PaCOMT1-PaCOMT4*) were extracted from the resulting transcriptome sequence set and aligned with known Class II OMT sequences using DNAMAN v7 software (Lynnon LLC, San Ramon, CA, USA). A phylogenetic analysis was performed based on the maximum likelihood method implemented in MEGA v4.0 software [27]. The 962 bp *PaCOMT2* sequence, lying at the 5′ end of the coding region, was used to generate three gene-specific primers denoted PaCOMT2-GSP-1/-2/-3 (Table S1) in order to amplify its 3′ end via RACE-PCR. A SMART RACE cDNA Amplification Kit (Clontech, Mountain View, CA, USA) was used for this purpose. The complete cDNA sequence was inferred from that of the partial cDNA fragments. The full-length sequences of *PaCOMT1* through *4* were amplified from *P. appendiculatum* using various gene-specific primer pairs (Table S2), A-T cloned into the pMD19-T vector (Takara, Otsu, Japan), then transformed into *E. coli* DH5α.

### 4.3. Heterologous Expression and Purification

The *PaCOMT1* cDNA sequence proved to be about 80 bp longer than that of a typical *COMT*. It harbored two start codons, resulting in two possible transcripts, denoted *PaCOMT1-Tr* and *PaCOMT1*. The corresponding open reading frames (ORFs), along with those from PaCOMT2 through 4, were amplified from the four cDNA clones using the gene-specific primer pairs given in Table S3. The resulting amplicons were digested with a number of restriction enzymes (Takara) and ligated into the pET32a vector (Novagen, Darmstadt, Germany) digested with the respective restriction enzymes. The constructs were transformed into *E. coli* strain BL21 (DE3), and the transgenic cultures were incubated at 37 °C until the OD_600_ reached 0.5. Expression of the transgene was induced by exposure to 1mM (80 μL) isopropyl β-d-1-thiogalactopyranoside (IPTG) for 16 h and a drop in the temperature to 18 °C. *N*-terminal hexahistidine-tagged proteins were purified by passing through a Ni-NTA Sefinose His-bind column (Bio Basic Inc., Markham, ON, Canada), and then were exchanged through an Ultrafiltration tube (Millipore, MA, USA) in the presence of binding buffer (20 mM Tris–HCl, 500 mM NaCl, pH 8.0). The homogeneity of the heterologously expressed protein was determined by SDS-PAGE and its concentration was assessed using Bradford’s reagent (Beyotime, Shanghai, China) employing bovine serum albumin as the standard.

### 4.4. Enzyme Assays

Enzyme assays were performed in 50 μL volume reactions, each containing 1mg/mL purified protein (2 μL), 400mM DTT (0.5 μL), 50mM SAM(0.5 μL), and 10mM substrate (1 μL), 400 mM Tris-HCl buffer (pH 7.5, 25μL) and double distilled water (25 μL). The reactions were incubated at 37 °C for 30 min and terminated by the addition of 50 μL of acetonitrile. When caffeoyl CoA was employed as substrate, enzyme assays were performed using 4-fold reaction volumes and stopped by adding 5 M NaOH (12 μL) to hydrolyze its thioester bond. To neutralize the mixture, 6 M HCl (28 μL) was added, and this mixture was afterwards extracted by an equal volume of ethyl acetate. The resulting extract was dried and then redissolved in 50 μL of methanol. Proteins extracted from *E. coli* BL21 cells harboring an empty pET32a plasmid were used as the negative control. Reaction products were subjected to LC-MS analysis, using an Agilent 1100 system (Agilent Technologies, Santa Clara, CA, USA) equipped with a reversed phase C18 column (Agilent) and an electron spray ionization mass spectrometer. The two mobile phases were 0.1% aqueous acetic acid and methanol. The flow rate was set to 1.0 mL/min, applying a linear gradient from 20% methanol to 50% methanol over 20 min for the phenylpropanoids, and from 35% methanol to 65% methanol for the flavonoids. The absorbance of the reaction product was monitored at 320 nm and 346 nm. The effect of varying the pH and temperature on enzyme activity was determined by comparisons with the standard assay conditions described above. The range of pH was 6.0–9.0 and the temperature range investigated was 25–50 °C. The impact of the presence of Mg^2+^ cations was assessed by replacing MgCl_2_ with either ethylene diamine tetraacetic acid (EDTA) or water. Kinetic assays were performed by varying the concentration of apigenin or luteolin from 5–400 μM at the optimal pH and temperature and allowing a 10 min reaction time. The quantity of reaction product generated was estimated using a standard calibration curve. Subsequently, *V_max_*, *K_m_*, and *K_i_* values were calculated using the Michaelis-Menten equation implemented in Graphpad Prism 5 software (GraphPad Software, La Jolla, CA, USA).

### 4.5. Production of Apigenin 4′-O-Methoxides in E. coli

*E. coli* BL21 cells harboring *pET32a-PaCOMT1* were pre-cultured overnight at 37 °C in 3 mL of LB liquid medium; the following day, 300 μL of the culture was seeded into 15 mL of LB liquid medium in the presence of the ampicillin antibiotic, and the culture was maintained at 37 °C until the OD_600_ had reached 0.6. IPTG was then added to a concentration of 0.4 mM, and the cells were held at 16 °C for 5 h. Then, apigenin dissolved in dimethyl sulfoxide was added at a concentration of 100 μM. After 12–48 h incubation at 16 °C, a 500 μL aliquot was collected and extracted in an equal volume of ethyl acetate, the extracts air-dried at room temperature, and the residue dissolved in 100 μL of methanol. For the subsequent HPLC analysis, extracts from *E. coli* BL21 cells carrying an empty pET32a plasmid were used as the negative control.

### 4.6. Accession Number

The Genbank accession numbers used are as followed: *Plagiochasma appendiculatum* transcriptome sequence (SRP073827), *Mesembryanthemum crystallinum* PFOMT (AY145521), PaF4′OMT (KY977687), PaCOMT2 (KY977688), PaCOMT3 (KY977689), PaCOMT4 (KY977690), *Plagiochasma appendiculatum* OMT1 (KP729179), *Medicago sativa* CCoAOMT (AAC28973.1), *Populus tomentosa* CCoAOMT (ACE95173.1), *Eucalyptus gunnii* CCoAOMT (CAA72911.1), *Broussonetia papyrifera* CCoAOMT (AAT37172.1), *Nicotiana tabacum* CCoAOMT (AAC49913.1), *Sorghum bicolor* COMT (AAL57301), *Zea mays* COMT (Q06509), *Arabidopsis thaliana* COMT (NP-200227), *Prunus dulcis* COMT (Q43609), *Populus tomentosa* COMT (AAF63200), *Medicago sativa* COMT (AAB46623), *Stylosanthes humilis* COMT (2119166A), *Medicago sativa* IOMT (AAC49927), *Glycyrrhiza echinata* D7OMT (AB091685), *Medicago truncatula* IOMT4 (DQ419912), *Hordeum vulgare* F7OMT (X77467), *Medicago truncatula* IOMT7 (DQ419914), and *Glycyrrhiz aechinata* HI4′OMT (AB091684).

### 4.7. Catalog Number

Catalog numbers are as follows: caffeic acid (A15950), SAM (A4377), apigenin (A0113), luteolin (A0108), scutellarein (A0779), daidzein (A0008), baicalein (A0018), acacetin (A0763), eriodictyol (A0402), naringenin (A0147), quercetin (A0083), kaempferol (A0129), and genistein (A0009).

## 5. Conclusions

In summary, PaF4′OMT is characterized as able to preferentially methylate the apigenin 4′-OH group to form acacetin. It is also the first Class II OMT to be isolated from a liverwort species. The enzyme was confirmed to be a highly site-specific OMT, able to exclusively methylate the flavone 4′-OH group in the presence of a methyl donor. This high selectivity provides a simple synthetic pathway for the bio-based transformation of apigenin and other flavones.

## Figures and Tables

**Table 1:** Specific activity of purified PaF4′OMT and PaCOMT1-Tr recombinant proteins with selected substrates.

Substrates	PaF4′OMT	PaCOMT1-Tr
Apigenin	86.84 ± 1.76^a^	11.30 ± 1.07
Luteolin	32.89 ± 2.63	5.26 ± 0.59
Scutellarein	14.26 ± 1.64	ND^b^
Genkwanin	7.77 ± 0.75	ND
Baicalein	ND	ND
Acacetin	ND	ND
Eriodictyol	1.61 ± 0.12	ND
Naringenin	<1	ND
Quercetin	7.60 ± 0.79	ND
Kaempferol	8.17 ± 0.50	ND
Genistein	2.21 ± 0.53	ND
Caffeic acid	ND	ND
Caffeoyl aldehyde	ND	ND
Caffeoyl alcohol	ND	ND
5-Hydroxyconiferyl aldehyde	ND	ND
5-Hydroxyconiferyl alcohol	ND	ND

^a^ Activities presented in nmol mg^−1^ min^−1^ ± STDEV. ^b^ No product detected.

**Table 2:** Kinetic parameters of recombinant PaF4′OMT using apigenin and luteolin as substrates.

Substrates	*K_m_* (μM)	*V_max_* (nmol mg^−1^ min^−1^)	*K_cat_* (s^−1^)	*K_enz_* (M^−1^ s^−1^)	*K_i_*(μM)
Apigenin	31.0 ± 5.90	162.5 ± 7.29	0.117 ± 0.005	3795.9	−
Luteolin	52.1 ± 10.54	110.8 ± 12.63	0.080 ± 0.009	1537.8	292.8 ± 69.03

## Supplementary Materials

Supplementary Materials are available online.
