# Supplementary material for: The Functional Characterization of a Site-Specific Apigenin 4′-O-methyltransferase Synthesized by the Liverwort Species Plagiochasma appendiculatum
Source: Molecules. 2017 May 7;22(5):759. doi: 10.3390/molecules22050759 (PMC6154639; doi:10.3390/molecules22050759)
Supplement: Supplementary file 1 [file molecules-22-00759-s001.pdf]

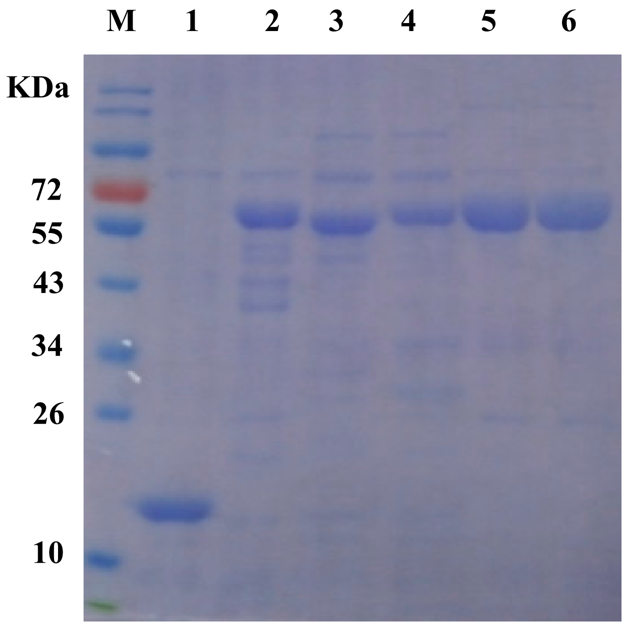

Figure S1. SDS-PAGE analysis of recombinant proteins. Each lane was loaded with the output of *E. coli* cells harboring a transgene construct. Lane 1: pET32a (empty vector control), lane 2: PaF4'OMT, lane 3: PaCOMT1-Tr, lane 4: PaCOMT2, lane 5: PaCOMT3, lane 6: PaCOMT4. M: molecular mass standards.

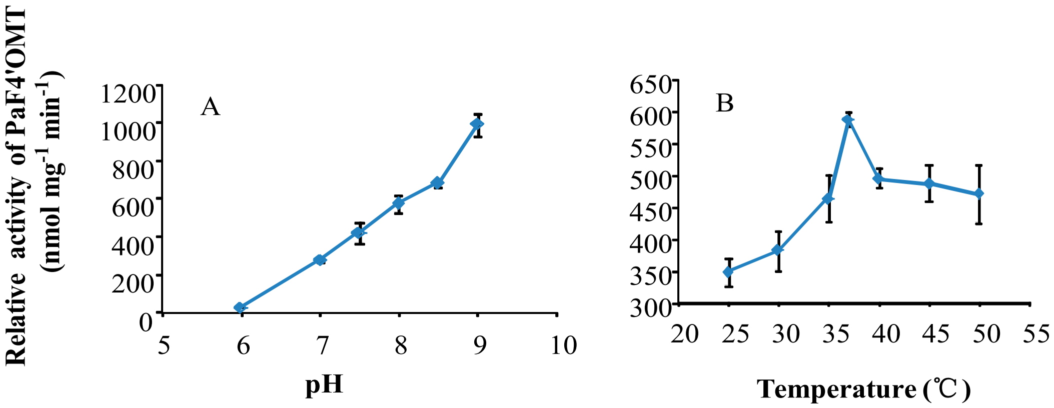

Figure S2. The effect of varying the pH and temperature on enzyme activity.

Table S1 Sequences of oligonucleotide primers used for 3' RACE.

Primer name	Primer sequences (5' to 3')
PaCOMT2-GSP-1	ACCCTGGGCACTGAAGGCTGTTGTGA
PaCOMT2-GSP-2	CGCCGTATCTATCCTGGAGCGCGTCA

PaCOMT2-GSP-3	TGGCCGTTCTCCACAACAACAGCGA

Table S2 Sequences of oligonucleotide primers used for cDNA cloning.

Primer name	Primer sequences (5' to 3')
PaCOMT1-F1	TATTGAGGACGCCGAGAG
PaCOMT1 -R1	CAGCGTAGAATGGATATG
PaCOMT2 -F1	CCTCCACATCGTCCATAGT
PaCOMT2 -R1	CAGAATACCAAGCAGCAAC
PaCOMT2 –F2	GCAGACGAGTGTGAGAGAC
PaCOMT3-F1	AAGCGGTCTGTTAGTGAC
PaCOMT3-R1	CGGGAGTTGTTGTCTGAG
PaCOMT4 -F1	CTCCAAAACTTCGAGCT
PaCOMT4 -R1	CAGTATCCTATGGCACC
PaCOMT4 - F2	TATTTGAGTGCTAGCCAG
PaCOMT4 –R2	GACATTTCGCCTCAGTGCT

Table S3 Sequences of oligonucleotide primers used for heterologous expression.

Primer name	Primer sequences (5' to 3')
PaCOMT1-PF	CGGGATCCATGGCGGTATCCACGAATGG
PaCOMT1-PR	CCCTCGAGCTACTTCCGGACCTCTATGA
PaCOMT1-Tr-PF	CGGGATCCATGTCTGTTAGCGCCAATGT
PaCOMT2-PF	CGGGATCCATGTCAATAGCCATGAGTGG
PaCOMT2-PR	CCCAAGCTTTCAGTGCTGAATTTTACGGA
PaCOMT3-PF	CGAGCTCATGGTCCCACAAGATCAAAG
PaCOMT3-PR	CCCTCGAGTCAAGACATGGGAACTTCGA
PaCOMT4-PF	GGAATTCATGGTCCCACAAGATCAAAG

PaCOMT4-PR	CCCTCGAGTCAAGACATGGGAACTTCGA
